# PAR1 is selectively over expressed in high grade breast cancer patients: a cohort study

**DOI:** 10.1186/1479-5876-7-47

**Published:** 2009-06-18

**Authors:** Norma A Hernández, Elma Correa, Esther P Avila, Teresa A Vela, Víctor M Pérez

**Affiliations:** 1 Subdirección de Investigación Básica, Instituto Nacional de Cancerología, Mexico City, Mexico; 2 Patología Post-Mortem y Tumores Mamarios, Instituto Nacional de Cancerología, Mexico City, Mexico

## Abstract

**Background:**

The protease-activated receptor (PAR1) expression is correlated with the degree of invasiveness in cell lines. Nevertheless it has never been directed involved in breast cancer patients progression. The aim of this study was to determine whether PAR1 expression could be used as predictor of metastases and mortality.

**Methods:**

In a cohort of patients with infiltrating ductal carcinoma studied longitudinally since 1996 and until 2007, PAR1 over-expression was assessed by immunoblotting, immunohistochemistry, and flow citometry. Chi-square and log rank tests were used to determine whether there was a statistical association between PAR1 overexpression and metastases, mortality, and survival. Multivariate analysis was performed including HER1, stage, ER and nodes status to evaluate PAR1 as an independent prognostic factor.

**Results:**

Follow up was 95 months (range: 2–130 months). We assayed PAR1 in a cohort of patients composed of 136 patients; we found PAR1 expression assayed by immunoblotting was selectively associated with high grade patients (50 cases of the study cohort; P = 0.001). Twenty-nine of 50 (58%) patients overexpressed PAR1, and 23 of these (46%) developed metastases. HER1, stage, ER and PAR1 overexpression were robustly correlated (Cox regression, P = 0.002, P = 0.024 and P = 0.002 respectively). Twenty-one of the 50 patients (42%) expressed both receptors (PAR1 and HER1 P = 0.0004). We also found a statistically significant correlation between PAR1 overexpression and increased mortality (P = 0.0001) and development of metastases (P = 0.0009).

**Conclusion:**

Our data suggest PAR1 overexpression may be involved in the development of metastases in breast cancer patient and is associated with undifferentiated cellular progression of the tumor. Further studies are needed to understand PAR1 mechanism of action and in a near future assay its potential use as risk factor for metastasis development in high grade breast cancer patients.

## Background

Breast cancer is a health problem, specifically in developing countries, where early diagnosis systems are lacking and mortality rates continue to increase. In Mexico up to 25 new cases of breast cancer are diagnosed everyday with mortality rates reaching 15.7 per 100,000 in women under 25 years of age [1,2]. Metastases to bone, lung, liver, and the central nervous system represent the main complication of treatment and also the main cause of death. For example, breast cancer patients with pulmonary metastases have an overall survival rate of 38% and 22% by five and ten years respectively after the initial cancer diagnosis [3,4].

Recent discovery of new factors involved in breast cancer progression *in vitro*, are difficult to translate into diagnostic tools to accurately identify patients at high risk of metastasis. To improve treatment and survival of these patients, a better molecular understanding of the early mechanisms leading to metastases is required [5,6]. The thrombin receptor, protease-activated receptor-1 (PAR1), participates in a variety of biological processes, such as tissue remodelling, inflammation, proliferation and angiogenesis. PAR1 has long been thought to be involved in tumour invasion, metastases associated with melanomas, as well as with cancer of the breast, colon, lung, pancreas, and prostate [7,8]. Although the exact role of PAR1 in tumour cell invasion is not completely understood, it is thought that PAR1 promote detachment and subsequent migration of epithelial cancer cells from and through the basement membrane, a key step in tumour metastases [9-14]. Normal breast epithelial cells do not have the capacity to migrate efficiently in response to chemotactic signals [9-14].

PAR1 is a G protein-coupled receptor. Four different PARs have been identified: PAR1, PAR2, PAR3, and PAR4. PAR1 and PAR3 are activated by thrombin, PAR2 is activated by tryptase or trypsin, and PAR4 is activated by both thrombin and tryptase or trypsin. PAR1, the prototype member of the PAR family, becomes activated when thrombin cleaves a specific residue sequence (R_41_-S_42_) within the receptor's N-terminal extracellular domain. Synthetic peptides that correspond to the first few amino acids of freshly cleaved N terminus (SFLLRN) can function as intramolecular agonists of PAR1. In several experimental models (*in vitro *and *in vivo*), it has been shown that thrombin enhances both tumour cell adhesion to extracellular matrix proteins and the number of lung metastases in animal models [11,12].

In established cancer cell lines, PAR1 expression levels correlate directly with the degree of cancer invasiveness. The human carcinoma breast cancer cell line MDA-MB-231, which is highly invasive, expresses very high levels of functional PAR1, PAR2, and PAR4. Another human carcinoma breast cancer cell line, MCF-7, which is minimally invasive, expresses only trace amounts of PAR1 and low levels of PAR2 and PAR4. These data are consistent with findings showing that high levels of PAR1 mRNA are found in infiltrating ductal carcinoma, whereas very low amounts are found in normal and premalignant atypical intraductal hyperplasia [13-16].

Despite these advances, the role of PAR1 in breast cancer cell invasion is not completely understood. It has been suggested that thrombin indirectly induces cellular rearrangements by activating PAR1 and transactivating the epidermal growth factor receptor (EGFR and/or HER2) poor prognosis factors for breast cancer patients, which exerts its effects exclusively through intracellular signals. PAR1 has been specifically shown to be involved in the migration and invasiveness of MDA-MB-231 cells via a Gi protein-phosphatidylinositol 3-kinase dependent pathway. Matrix metalloprotease-1 is responsible for activating the invasive functions of PAR1 [[13,14,17] and [18]].

Taken together, these findings prompted us to investigate the role of PAR1 in the development of metastases in breast cancer patients. Our aim was to determine whether PAR1 expression patterns in patients diagnosed with infiltrating ductal carcinoma correlate with long-term clinical outcome. Development of metastases in these patients was used to determine the biologic aggressiveness of the cancer. We believe that cellular factors associated with poor outcome, such as EGFR, HER2 and PAR1 overexpression, if associated with metastases or mortality, could serve to identify patients at high risk to develop metastatic breast cancer. We found significant correlations between PAR1 overexpression and development of metastases and increased mortality. Our data suggest that PAR1 plays an important role in the development of metastases in breast cancer patients. Further studies at the cellular level are essential to clarify the precise role of PAR1 in breast cancer patient's progression.

## Methods

### Patients

A cohort study was undertaken on a group of 136 female patients from our Institution. They were admitted during first three months of 1996 with a diagnosis of infiltrating ductal carcinoma of the breast; inclusion criteria was limited to women virgin of any treatment elsewhere and confirmed diagnosis of ductal carcinoma; they were followed longitudinally until 2007. After approval from our Institutional board, and with a signed informed consent from each patient, in all cases tissue blocks were taken from the original biopsy used for diagnosis and prior any treatment for PAR1 determination.

The demographic, clinical, and pathological variables examined were age, age at menarche, age at first birth, parity, breastfeeding (considered positive, if were sustained for more than 3 months), clinically and surgical positive axillaries nodes, hormonal status and tumor size. The pathologic size was determined after surgery based upon the greatest dimension of the macroscopic specimen. All patients were infiltrating ductal carcinoma for histological type with a SBR ≥5–9. Classification of the histological type and SBR were made by review of all available histological material by two independent pathologists, who determined the diagnosis and determined tumour grade according to Elston classification [19].

First diagnosis of metastases was noted as the time to first appearance. In all cases diagnosis of metastases was confirmed by X-ray and/or CT-scan for lung metastasis, gamma gram for bone metastases, ultrasonic detection or CT-scan for Liver and CT-scan or magnetic resonance for CNS. Up to four metastases sites were considered; we have not collected tissue samples from all metastasis developed in our cohort patients. Survival was recorded from time of diagnosis to dead. The follow up period began at the date of diagnosis. Patients were followed until death or censored from this analysis at the time of their last visit to our Institution.

### Immunoblotting

We used a 50 μm thick sample from each patient, taken from serial paraffin sections. All samples were evaluated by two independent pathologists; if necrosis or positive margins were present the cases were not included in the study. After paraffin removal, tissues were lysed using a collagenase and trypsin buffers over night at 37°C and suspended in lysis buffer (20 mM Tris HCl pH = 7.8, 50 mM NaF, 40 mM Na_4_P_2_O_7_, 5 mM MgCl_2_, 10 mM Na_3_VO_4_, 1% triton X-100, 0.1% SDS and 5 mM Benzamidine) supplemented with 1 μg/ml each of pepstatin, leupeptin, aprotinin and 2 μM phenyl methyl sulphonyl fluoride (PMSF). After incubation of 20 minutes at 4°C, and removal of cell debris, lysates were centrifuged at 15,000 g for 15 min at 4°C. Clear lysates were separated by SDS-polyacrylamide gel electrophoresis (SDS-PAGE 12%), blotted into a PVDF membrane (Amersham Life Science) followed by immunoblotting to assess PAR1. As a positive control we also assayed EGFR and HER2 expression, both are well known poor prognostic factor for the outcome of metastatic breast cancer patients, but also known as downstream mediators of PAR1 activation [17,18].

We used a 1:2000 dilution of a mouse monoclonal antibody raised against aminoacids 42–45 of thrombin receptor of human origin (Santa Cruz Bio-Technology). And we also used an anti-EGF receptor mouse monoclonal antibody at same dilution (Upstate) which recognizes the motif NAEYLR of the EGFR from mouse and human origin. Anti-rabbit polyclonal antibody raised against HER2 receptor from human origin (upstate) was also used at 1:1000 dilution. As a loading control we performed an immunoblot using a 1:2000 dilution of a polyclonal antibody directed against Glyceraldehyde-3-phosphate dehydrogenase (GAPDH) clone V-18 from Santa Cruz Bio-Technology.

Enhanced Chemoluminescence was used to develop the membranes (Amersham Life Science). PVDF membranes were used in all cases (Amersham Life Science). Quantification of the expression of the different mediators was calculated with Aida software and presented as experimental value - control value/control value × 100 where the control value was derived from lysates of cells mock exposed.

In order to validate and give strength to our results we used two different human breast cancer cell lines as positive (MDA-MB-231) or negative (MCF-7) control for PAR1 and EGFR expression as previously reported ([13], data not shown).

### Immunohistochemistry (IHC)

IHC staining was carried out for PAR and HER2. We used an antibody that recognizes the N-terminal extracellular loop of human thrombin receptor by immunohistochemistry with formalin-fixed, paraffin-embedded tissues (Sigma); we also used a polyclonal antibody that recognizes amino acids 1243–1255 from the human c-erbB-2/HER2 (Upstate). We compared data obtained by IHC versus that one obtained by western blotting. Method was described previously [20]; briefly the tissues were fixed in 10% buffered formalin, processed and embedded in paraffin. Section 3-μm thick were then cut and dried for 12 h at 37°C. One section from each block was stained with H&E. The sections were de-paraffinised in xylene and re-hydrated through graded concentrations of ethanol to distilled water. Incubating the sections in methanol and hydrogen peroxidase for 30 minutes quenched endogenous peroxidase. Immunohistochemical staining was performed by using the ABC system (Bio Genex, CA USA) and DAB as substrate. Blocking serum was applied and incubated for 15 minutes. Then we started the incubation with the primary antibody diluted 1:500 for each antibody. Sections were incubated with the biotinylated secondary antibody and were developed using the peroxidase substrate.

Each staining run included both positive and negative control slides. The positive control slide was prepared from tissue known to contain HER2; the negative control slide was prepared from the same tissue block as the specimen, however instead of using a primary antibody, this one was incubated with an isotype-matched antibody. HER2 staining was scored utilizing a 3-point scoring system; we considered positive staining, if we observed strong continue and intense staining of the membrane in more than 10% of the cells in the slide. PAR1 were scored positive if any (weak or strong) cytoplasmic and/or membranous invasive carcinoma cell staining was observed in more than 10% of the cells in the slide. Slides were evaluated for two different pathologists.

### PAR1 Immunofluorescent staining by Flow Citometry (IF)

To determine PAR1 expression in paraffin-embedded sections from breast cancer patients we used same antibody for western blotting. Tissue samples from the patients were disaggregated into single cell suspensions (collagenase and trypsin 0.25%). Cells (1 × 10^6^) were probed with 1:500 anti-PAR1 dilution of the mouse monoclonal antibody rose against amino acids 42–45 of thrombin receptor of human origin (Thrombin R; ATAP2, Santa Cruz Bio-Technology); and then treated with a goat anti-mouse IgG (H+L) fluorescein conjugate (goat polyclonal). FITC labelled cells were analyzed by flow citometry.

### Statistical Analysis

The Chi-square or Fisher tests were used to determine differences between proportions. Overall survival was obtained by the PAR1 estimates by Kaplan-Meier method, and differences between distributions were evaluated by the log-rank test. A Cox Regression was performed including clinical stage, Oestrogen receptor alpha and lymph node status to evaluate PAR1 potential as an independent prognostic factor. P values equal or less than 0.05 was considered statistically significant.

## Results

### Over expression of PAR1

**Figure 1:**
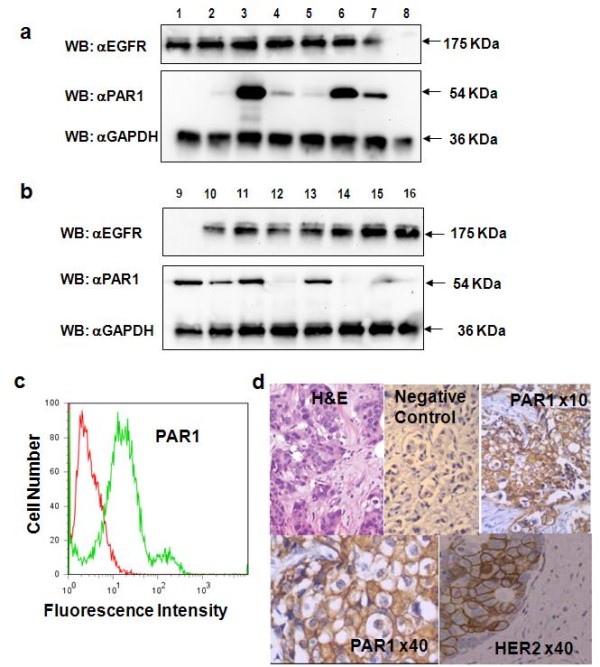
**PAR1 expression in breast cancer patients**. Western blots showing PAR and EGFR expression profiles of tumor biopsy samples from patients with infiltrating ductal carcinoma (Figure 1a and 1b). The blots are representative of three replicate tests. (c) A representative example of immunofluorecent staining of PAR1; Red line: background fluorescence (secondary antibody alone); green line: fluorescent shift attributable to PAR1 expression. Traces shown are representative of one of three independent measurements. (d) A tissue sample exhibiting PAR1 (visualized using ×10 and ×40, objective lens) and HER2 strong membrane immunostaining; also shown: H&E and a negative control sections.

We assayed PAR1 expression in all samples (136 cases of ductal carcinoma) of the study cohort by IHC, IF and western blotting; however, we found PAR1 receptor expression only in those patients with high grade. Negative results are not shown and we are presenting data from the high grade cases we included in our cohort (50 cases). The median follow-up time of the patients included in the present study was 95 months (range: 2–130 months). Western blot analysis of biopsy samples revealed that 29 of 50 (58%) patients with infiltrating ductal carcinoma of the breast, expressed PAR1 (Figure 1a and 1b, Table 1). Densitometric quantification of PAR1-immunoreactive bands indicated that 20 of the 29 patients (69%) overexpressed PAR1 by more than 70% (Figure 1a and 1b) of that expressed by a mock exposed invasive breast cancer cell line (previously described in methods, data not shown). Twenty one of 50 (42%) high grade patients did not express PAR1 at all (Table 1 and Figure 1). We also confirmed PAR1 expression in samples from our patients using immunofluorescent staining for PAR1 present in the surface of the cells (Figure 1c), and found 30 patients out of 50 high grade breast cancer patients included in this study (70%) express PAR1; highly significant when compared with the rest of the group (P = 0.0001).

**Table 1:** PAR1 expression in breast cancer patients

**Western blotting PAR1 immunoreactivity**	No. of patients* (%)	**Patients with metastases^**†**^(%)**
Positive	29/50 (58%)	23/23 (100%)
Negative	21/50 (42%)	0

*Total number of patients (N_T _= 50)

^†^Number of patients with metastases (N = 23/50 [46%])

In regard to IHC data, as expected, more samples showed PAR1-immunoreactivity by immunoblotting than the ones assayed by IHC (Figure 1d); we found 25 samples positive for PAR1 expression (50%) by IHC. Nevertheless, all samples showing PAR1-immunoreactivity with IHC were also positive when assayed by Western blotting. Spearman correlation between PAR1 expression measured by IHC versus that measured by immunoblotting was highly significant (P = 0.0005, r = 0.4767). Our analysis was carried out using the more sensitive and quantitative immunoblotting results, but it is important to mention, that all 25 tumor samples positive for PAR1 expression shown different degree of immune reactivity: 48.3% stained lightly, 24.1% moderately and 27.6% strongly. PAR1 expression was found mainly in the entire membrane although some cytoplasmatic staining was also observed (Figure 1d); we found some degree of variation in the staining of PAR1 within the tumor; although we were assaying a biopsy sample of the tumor; we have been able to assayed some tumor samples (from the surgery), initially found PAR1 positive; roughly we found more than 50% of the tumors cells were immune reactive for PAR1 staining; non significant staining was found in the surrounding tumor microenvironment. (Figure 1d) Regarding HER2 expression we found 5% tumors tested were HER2 negative, 38.3% stained weakly, 34.8% moderately and 28.2% strongly.

### Correlations between HER2 and EGFR and PAR1 over expression

To determine whether there is an association between HER2, EGFR1 and PAR1 expression, we assessed the expression of these markers in biopsy tissue obtained from our patients (Figure 1). Twenty-five of 50 (50%) samples expressed EGFR1 and HER2. Densitometry analysis revealed that 23 of the 25 (92%) samples overexpressed EGFR1 and HER2 by more than 80% compared to controls (breast cancer cell lines as previously described; data not shown). Twenty-one of 50 (42%) samples expressed both PAR1 and EGFR1 and HER2. Statistical analysis revealed a significant correlation between PAR1 and EGFR1 overexpression (Fisher exact two-tailed test, P = 0.0004; Spearman Rank correlation, r = 0.5755, P = 0.0001). To evaluate the diagnostic potential of EGFR1 expression in relation to PAR1, we also measured the sensitivity, specificity, and predictive powers of this association. We found a sensitivity value of 0.72 (95% confidence interval: 0.53 to 0.87), a specificity value of 0.81 (0.58 to 0.95), a positive predictive value of 0.84 (0.64 to 0.95), and a negative predictive value of 0.68 (0.46 to 0.85). In summary, if the breast cancer sample expressed EGFR1, it was likely to also express PAR1.

### PAR1 over expression and metastases development

**Figure 2:**
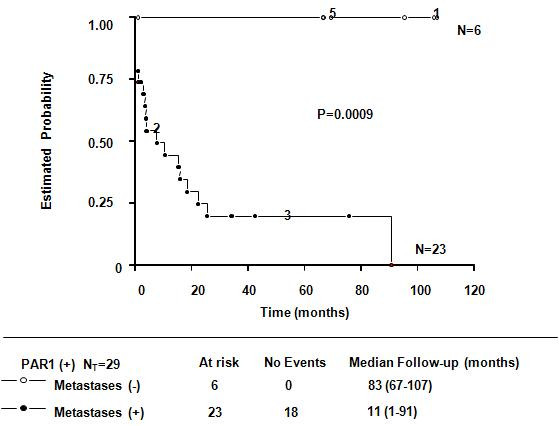
**Kaplan-Meier survival estimates of breast cancer patients overexpressing PAR1: those with and without metastases**. The survival of high-grade breast cancer patients overexpressing PAR1 (N = 29) is shown as a function of metastases development. The differences between overall survival distributions were statistically significant (P = 0.0009).

Disease progressed rapidly in our study population (Figure 2). Of the 50 patients assessed, 23 (46%) developed metastases, mostly within the first 24 months after receiving their cancer diagnosis. We found a significant association between PAR1 overexpression and metastases: all 23 of these patients overexpressed PAR1 (Table 1, Figure 2). Comparing this group with the group that did not develop metastases and did not overexpress PAR1, Fisher's exact test and a log rank test revealed a highly a reliable difference (P < 0.0001 and P = 0.00009, respectively). Although 23 of 29 (79%) of the patients overexpressing PAR1 developed metastases during the study, it is notable that 10 (35%) of these patients already had at least one metastasis at the beginning of this study, indicating the advanced clinical status of our patients. We also found a significant correlation between EGFR1 overexpression and metastases development in our patients (P < 0.0001, data not shown). Also it was of interest to analyse the distribution of metastases by organ and the order of appearance on a patient-by-patient basis. We found that 22 of 50 (44%) patients developed their first metastasis in the following locations: 10 of 22 (45%) in extra-axillary lymphatic nodules, 6 of 22 (27%) in bone, 5 of 22 (23%) in lung and 1 of 22 (5%) in liver.

### PAR1 overexpression and mortality

**Figure 3:**
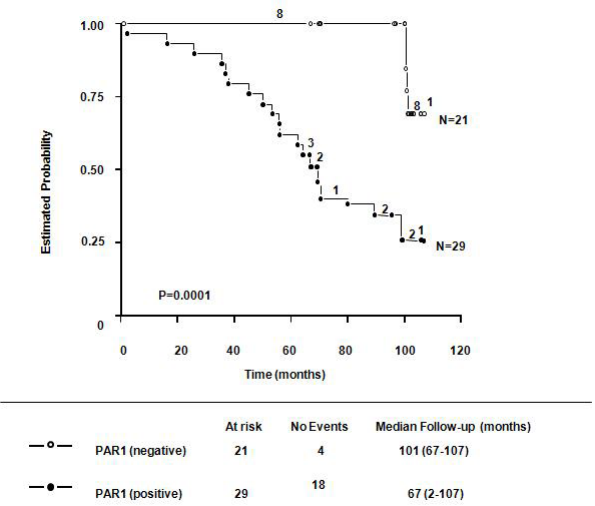
**Overall Kaplan-Meier survival estimates as a function of PAR1 expression**. The overall survival of breast cancer patients is shown according to PAR1 overexpression. The differences between overall survival distributions were statistically significant (P = 0.0001).

Twenty-two of 50 patients (44%) died of their disease. Eighteen (36%) expressed PAR1. That is, of the 29 PAR1-positive patients participating in our study, 18 (62%) died. Fisher exact test analysis revealed a statistically significant link between PAR1 overexpression and increased mortality (P < 0.0001). This link was also supported by Kaplan-Meier overall survival analysis (Figure 3). Differences between overall survival distributions were highly significant as determined by a log-rank test (P = 0.0001). We also found a significant correlation between EGFR overexpression and mortality (P < 0.0001, data not shown). In addition to examining positive association factors, we also analyzed our group of patients for usual prognostic factors associated with tumour mortality in breast cancer. Tumour size (≥5) and presence of pulmonary metastases were significantly correlated, specifically in groups of patients with or without PAR1 over-expression (P = 0.0004 and P = 0.0012 respectively).

### Differences in demographic and clinical variables among PAR1-positive/negative patients

To determine the potential of PAR1 as a useful prognostic factor for breast cancer patients, independent of the prognostic factors for tumour mortality; we compared the demographic, clinical, and pathological characteristics of the 29 PAR1-positive patients to those of the 21 PAR1-negative patients (Table 2). We found no major differences between the two groups regarding age, age at menarche, age at first birth, parity, or breast-feeding. Moreover, we found no significant differences between the two groups regarding the following clinical and pathological parameters: the number of affected lymphatic axillary nodules (surgically identified [data not shown] or clinically palpable), hormonal status, or tumour diameter.

**Table 2:** Distribution of demographic, clinical, and pathological variables of breast cancer patients as a function of PAR1 expression

**Variable**	PAR1*(+)	PAR1* (-)
Age (years)	50 (23–77)	47 (31–58)
Age at menarche (years)	13 (11–16)	13 (12–15)
Age at first birth (years)	23 (18–34)	22 (19–33)
Parity	3 (0–12)	2 (0–8)
Breastfeeding (>3 months)	15/29 (52%)	14/21 (67%)
		
Clinically positive axillary nodes	19/29 (66%)	15/21 (71%)
Hormonal status^†^		
Pre-menopausal	15/29 (52%)	11/21 (52%)
Post-menopausal	14/29 (48%)	10/21(48%)
		
Tumour diameter (cm)	7 (2–25)	6 (2–8)
		
ER^‡ ^(+)	15/29 (52%)	10/21 (48%)

Total (N_T _= 50)	29	21

*Median (range or percentage) unless specified.

^†^Circulating estrogen and progesterone levels

^‡^Estrogen receptor status

However, we did find a significant correlation between PAR1 status and cancer invasiveness (P < 0.05). The disease of patients with PAR1-positive tumours tended to be more clinically advanced than that of PAR1-negative patients. Of the 29 patients who were over expressing PAR1, 22 (76%) had IIIA-, IIIB-, or IV-stage breast cancer. Only seven of 29 (24%) had I-, IIA-, or IIB-stage cancer. In contrast, of the 21 PAR1-negative patients, only six (29%) had IIIA-stage or greater cancer, whereas 15 (71%) had IIB-stage or lower cancer.

We also performed a multivariate analysis including stage, estrogen receptor (alpha), and lymph node status to evaluate PAR1 as an independent prognostic factor. Although the small size of our cohort of patients, Cox regression demonstrates highly significant p values for EGFR (P = 0.002), stage (P = 0.024), and absence of estrogen receptor (P = 0.002). We did not find any significance for lymph node status (P = 0.441).

### Therapeutic treatment received by our patients

Our institution offers a diverse regimen of breast cancer treatments that can impact disease outcome, particularly the outcome of those in advanced stages of the cancer. To determine whether our treatment schemes had contributed to our finding that PAR1 status affects the clinical status of breast cancer, we grouped our patients by the treatment they received and carried out statistical analyses. All 50 high grade cases underwent radical resection of the tumor. The patients received both systemic and local therapy: systemic chemotherapy (mainly combinations of doxorubicin [Adriamycin^®^] and cycloheximide) and/or hormonotherapy (taxanes); and local chemotherapy and/or radiotherapy before surgery). PAR1-positive and PAR1-negative patients were treated similarly. There were no significant differences in the types of therapy received by PAR1-positive and PAR1-negative patients.

## Discussion

In the present study, we demonstrated that PAR1 overexpression assayed by immunoblotting is associated with an increased risk of metastases development and mortality in patients with breast cancer (Table 1, Figures 1, 2, 3). All patients with metastases overexpressed PAR1 (Figure 2). Moreover, the majority of our PAR1-overexpressing patients died during the course of this study (Figure 3). Our data suggest PAR1 plays an important role in the mechanisms underlying the development of metastases [[8,10], and [14]]. Our findings are consistent with previous findings showing mRNA of PAR1 is expressed in primary breast cancer tissue; mediates the invasive potential of certain breast cancer cell lines [13,15], and that it is involved in the tumour progression [16].

We also found a significant correlation between the co-overexpression of PAR1, EGFR1 and increased risk for metastases (Figure 1 and 2). This link is not surprising, since EGFR is a very well known poor prognostic factor in breast cancer patients [[8,21] and [22]]. Furthermore it had been shown that proteolytic activation of PAR1 by thrombin induces persistent transactivation of EGFR and ErbB2/HER2 in invasive breast carcinoma (23). Selectivity of PAR1 expression in tumor samples, its invasive potential shown in breast cancer cell lines, and the important role played by EGFR/HER2 as downstream transactivators of PAR1, indeed explains the positive correlation we found, between the expression of prognostic factors conveying poor disease outcome and poor tumour differentiation [15,16,24]. Furthermore in our experience, PAR1 it is not expressed at all or expressed at very low levels in tumor samples from breast cancer patients with SBR = 8 as previously assayed in our laboratory (data not shown). To treat high risk population effectively and as early as possible during the course of their disease, we need a better understanding of the mechanisms underlying tumour progression.

Although the significant correlation between PAR1 overexpression and increased mortality may be just a consequence of tumour progression translated as the establishment of metastases (Figures 2 and 3), this link is still significant. It is well documented that visceral (lung, liver) or CNS metastases result in the poorest prognostic outcome for any given cancer [3,25-27]. PAR1 has been shown to mediate the formation of pulmonary metastases in animal models of cancer [12]. In the present study, we found a very robust, significant correlation between PAR1 overexpression and the formation of pulmonary metastases. Most of the patients developed their first metastases within the first 24 months of being diagnosed; and the site of these metastases tended to affect extra-axillary nodules. Secondary metastatic sites were bone, lung, liver, and CNS. Taken together, these findings implicate PAR1 as a potential marker for aggressive cancer.

Our findings strongly implicate PAR1 as a prominent factor involved in tumour progression in breast cancer, thereby supporting its use as potential prognostic factor for invasive breast cancer. Indeed, we found that the clinical status or stage of breast cancer in our patients was correlated with PAR1 overexpression: patients overexpressing PAR1 in biopsy samples had more advanced disease than did patients not expressing PAR1. This may have very important implications at the cellular level [28,29], since PAR1 may be first expressed in high-grade patients when tumour progression is initiated. Thus, regular tracking of PAR1 status may be useful to identify early on breast cancer patients at high risk for metastases.

Furthermore we have demonstrated despite the small size of our cohort of patients, the multivariate analysis we performed, shown highly significant p values for EGFR (P = 0.002), stage (P = 0.024), and absence of estrogen receptor (P = 0.002). Our data strongly suggest PAR1 may be an independent prognostic factor for breast cancer patients. Although our sample of patients was very small, we are confident that PAR1 is an equally accurate prognostic factor for metastases and mortality as are EGFR and HER2 [21,22,24], since comparison of PAR1-positive and PAR1-negative patients revealed no significant differences in the main prognostic parameters typically considered in breast cancer (e.g., age, tumour diameter, hormonal status, etc).

Although treatments were diverse, we analysed this factor and found no remarkable differences between treatment types given to patients who showed PAR1 overexpression and those who did not. Our data identifies PAR1 as a potential prognostic factor for infiltrating ductal carcinoma. Its consistent involvement in the progression of breast cancer makes it an ideal prognostic tool only assayed in cell lines [30,31]. However, because our findings were based on a small sample of patients, the utility of PAR1 as a prognostic tool must be further assessed in a larger population of breast cancer patients, preferably through prospective studies. We are currently conducting further research to determine whether PAR1 can be used as an independent prognostic factor of these kinds of metastases in breast cancer patients with infiltrating ductal carcinoma. If our hypothesis about PAR1 is correct, the determination of PAR1 status can aid physicians in providing better follow-up therapy for these patients. Those at high risk for metastases can be identified early, allowing enough time for additional chemotherapy or surgical resection of metastases with the aim of achieving long-term survival or a longer disease-free period following surgical resection. This would improve the overall survival of high-grade breast cancer patients.

## Conclusion

Our data suggest PAR1 is involved in the development of metastases; showing a great potential as predictor of metastases and mortality in high grade breast cancer patients. Proteases have been implicated in tumor progression but PAR1 may be a good example of protease effectors implicated in tumour invasion and metastasis development and in a near future, PAR1 could become an ideal candidate for assessing new targets for drugs in the early diagnosis and treatment of metastasis in breast cancer patients.

## Competing interests

The authors declare that they have no competing interests.

## Authors' contributions

All authors (NAH, EC, EPA, TAV, VMP) had read and approved the final manuscript

NAH has made substantial contributions to the conceptions, design, analysis and interpretation of the data; she also help in the experimental performance of PAR1 detection and has been involved in drafting the manuscript. EC has made selection of the patient's cohort and reviewed all patient's charts, she also has made substantial contributions to the analysis and interpretation of the data. EPA has been involved in PAR1 detection (WB, IF and IHC), and has been participated actively in the analysis and interpretation of the data. TAV has been involved in the analysis of the immnuohistochemistry data, and help with the interpretation of the data. VMP Also have been involved in the analysis and interpretation of the immunohistochemistry data
